# Doxorubicin and *Trifolium pratense* L. (Red clover) extract synergistically inhibits brain and lung metastases in 4T1 tumor‐bearing BALB/c mice

**DOI:** 10.1002/fsn3.1820

**Published:** 2020-09-01

**Authors:** Mohsen Akbaribazm, Mohammad Rasoul Khazaei, Fatemeh Khazaei, Mozafar Khazaei

**Affiliations:** ^1^ Student Research Committee Kermanshah University of Medical Sciences Kermanshah Iran; ^2^ Fertility and Infertility Research Center Health Technology Institute Kermanshah University of Medical Sciences Kermanshah Iran

**Keywords:** 4T1, breast cancer, *GATA‐3*, isoflavone, metastasis, *Trifolium pratense* L.

## Abstract

*Trifolium pratense* L. (Red clover—*T. pratense*) commonly consumed as a healthy beverage has been demonstrated to have various biological activities including antioxidant and anticancer effects. The aim of this study was to investigate the antimetastasis effects of doxorubicin (DOX) and *T. pratense* extract in 4T1 tumor‐bearing BALB/c mice. In this study, 56 female BALB/c mice were randomly divided into seven groups (*n* = 8/group) to receive DOX and *T. pratense* extract in three different doses (100, 200, and 400 mg/kg/day) for 35 days. On day 36 after starting treatments, serum cytokines (IL‐8 and IL‐6) were measured. Immunohistochemical (IHC) staining was performed for GATA‐3 in the brain and lung, and for CK5/6 in tumor tissues. Metastasis‐related gene (matrix metalloproteinase‐2 [*MMP‐2*] and sirtuin‐1 [*SIRT‐1*]) expressions were also measured by real**‐**time PCR. Our results showed that cotreatment with DOX and *T. pratense* extract improved stereological parameters (i.e., reduction in the volume of metastatic tumors) in the lung and brain and decreased the serum levels of inflammatory cytokines (IL‐8 and IL‐6). DOX and *T. pratense* extract synergistically down‐regulated *MMP‐2* and up‐regulated *SIRT‐1* genes, decreased the number of CK5/6‐positive cells in tumor tissues, and inhibited metastasis of GATA‐3‐positive cells into the lung and brain. The combination of *T. pratense* extract and DOX synergistically inhibited the metastasis of 4T1 xenograft cells in a dose‐dependent manner.

## Graphical abstract

Doxorubicin and *Trifolium pratense* L. (Red clover) extract synergistically inhibited the metastasis of 4T1 to the lungs and brain by reducing cytokines (IL‐6 and IL‐8) and genes (SIRT‐1 and MMP‐2) associated with metastasis in tumor‐bearing BALB/c mice.

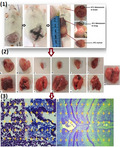

## 1 INTRODUCTION

Breast cancer (BC) is the most common malignancy in women with 18% prevalence and 1 million new cases each year. This cancer showed the highest mortality in the United States and claimed a mortality rate of 89.2 per 100,000 in the world in 2000 (Aurit, Devesa, Soliman, & Schairer, 2019). BC tumors present a heterogeneous phenotype regarding the expressions of different cellular receptors (estrogen receptors [ER], progesterone receptor [PR], and epidermal growth factor receptor 2 [HER2]) in tumor cells. Studies showed that 10%–20% of BC patients have triple‐negative phenotype, and 30% of BC tumors are estrogen‐independent (Bazm, Naseri, & Khazaei, 2018).

Regarding cellular origin, breast tumors are divided into two general categories: basal and luminal, and based on surface markers, they are divided into basal A, basal B, luminal A, and luminal B groups each demonstrating its own tumorigenic and metastatic properties (Bozorgi, Khazaei, & Khazaei, 2015). The 4T1 cell line shows a triple‐negative phenotype and lacks surface expressions of ER, PR, and HER2. The cell line is isolated from BALB/c mice with breast tumors and is resistant to hormone therapy and therapeutic monoclonal antibodies (Mohammadi, Zangeneh, Zangeneh, & Haghighi, 2020). The tumors created by this cell line can metastasize to various body tissues including the lung, bone marrow, brain, liver, and lymph nodes and only respond to chemotherapy drugs which usually have numerous side effects. Today, the latest treatment strategy against triple‐negative breast tumors is using chemotherapeutics in combination with dietary supplements (especially herbal compounds) to reduce side effects and promote drug synergism (DuPre, Redelman, & Hunter, 2007; Ma et al., 2020).

The most common metastatic target sites of triple‐negative breast cancer (TNBC) are the lung, bone, and liver. Approximately 60% of metastatic BC patients suffer from either lung metastasis or brain metastasis. Studies show that 60%–70% of the patients who die from metastatic BC are diagnosed with lung cancer as well (Tauro & Lynch, 2018).

There are various pathways involved in the development of distant metastasis in BC, including ROS/MAPK/AP‐1/MMP‐2 and IL‐6/STAT‐3/MMP‐2 pathways. Accordingly, it seems that MMP‐2 activity is necessary for successful metastasis of primary BC cells to a secondary site (Kim et al., 2018). In another study, Kim, Lee, Jeon, Lee, and Nam (2016) showed that the metastasis rate of TNBCs and the expressions of metastasis‐related proteins such as MMP‐2 and MMP‐9 increased after treatment with IL‐8 (Kim et al., 2016). Sirtuin‐1 (SIRT‐1) is a family member of NAD^+^‐dependent histone deacetylases, which has been shown to regulate cancer cells' metabolism, cell cycle, and metastasis (Liu, Zangeneh, Zangeneh, & Guo, 2020; Zhou, Yang, & Li, 2019). Although the role of SIRT‐1 in the regulation of cell cycle, metastasis, and differentiation of tumor cells has been studied in some cancers, its role in other types of cancer has not been well evaluated. SIRT‐1 was shown to inhibit the proliferation and metastasis of gastric cancer cells through activating the STAT3/MMP‐13 signaling pathway by inducing phosphorylation/acetylation of matrix metalloproteinase 13 (MMP‐13) and STAT3 in both in vivo and in vitro models (Zhang et al., 2019).

Doxorubicin (DOX) belongs to the family of anthracyclines anticancer drugs. It is a derivative of Streptomyces bacteria, especially Caesius strain and Peucetius variant. The drug delivers a success rate of 40%–50% in treating BC tumors, reaching to 60%–80%, if being used in combination with other effective drugs and compounds (Zangeneh, 2020). Inhibiting topoisomerase II, intercalating with DNA, and inducing free radicals are principal actions of anthracyclines against BC cells (Swift, Rephaeli, Nudelman, Phillips, & Cutts, 2006). Previous studies showed that resistance of BC cells to DOX is mediated by extracellular matrix (ECM) proteins. Various drugs and supplements (e.g., curcumin, quercetin, and selenocystine) can dramatically improve the safety profile and decrease the side effects (thrombocytopenia, cardiotoxicity, gastrointestinal disturbances, stomatitis, acute nausea and vomiting, and bone marrow aplasia) of DOX. Furthermore, the synergistic effects of supplementary agents can decrease drug resistance by inhibiting ECM proteins and regulating other cellular pathways (Hemmati, Joshani, Zangeneh, & Zangeneh, 2020; Hemmati, Zamenian, Delsooz, Zangeneh, & Mahdi Zangeneh, 2020; Staedler, Idrizi, Kenzaoui, & Juillerat‐Jeanneret, 2011; Zangeneh, Norouzi, Mahmoudi, Goicoechea, & Jalalvand, 2019).

*Trifolium pratense* L. (Red clover—*T. pratense*) belongs to fabaceae plant family and has a variety of therapeutic (such as expectorant, antiseptic, analgesic, sedative, disinfectant, anticoagulant, and febrifuge) properties. The plant extract has been described to improve pneumonia, meningitis, skin inflammation, and some reproductive and neuronal disorders (Booth et al., 2006). In our previous study on flavonoid and isoflavone compounds by high‐performance liquid chromatography–electrospray ionization–mass spectrometry (LC‐ESI‐MS), we showed that the leaves of this plant contained formononetin, genistein, biochanin A‐7‐glucoside, daidzein 7‐O‐β‐D glucoside, and biochanin A isoflavones, as well as 3‐O‐(Z)‐p‐coumaroylquinic acid, epigallocatechin, caffeic acid, quercetin‐3,7‐diglucoside, apigenin‐7‐O‐glucoside gallocatechin, and apigenin 6,8‐diglucoside flavonoids (Akbaribazm, Khazaei, & Khazaei, 2020a, 2020b).

Phytoestrogens are analogues of estrogenic compounds and can bind to both α‐ and β‐estrogen receptors (ERα and ERβ) with variable affinities (genistein > daidzein > biochanin A > formononetin) in various body tissues including the breast and endometrium (Miller, Collini, & Harris, 2003). These compounds are partial agonists of estrogen and have antiestrogenic roles in premenopausal period and weak estrogenic effects after menopause (e.g., dilatation of the ducts and germination of mammary glands). Isoflavones which present in the plant do not sprout in the ducts of mammary glands (Adlercreutz, 2002). Given the side effects of chemotherapy drugs and the need to reduce their doses which subsequently compromises their therapeutic effects, and considering the inhibitory effects of isoflavones on cancer metastasis, the present study aimed to investigate the antimetastatic effects of DOX in combination with red clover extract in 4T1‐induced BC bearing BALB/c mice.

## 2 MATERIALS AND METHODS

### 2.1 Extract preparation

The seeds of red clover (obtained from the Seed and Plant Improvement Institute) were sown in the research farm of Kermanshah University of Medical Sciences. The plant was identified and authenticated by a botanist and taxonomist in the Agricultural Research, Education and Extension Organization (AREEO), Karaj, Iran (voucher no: KPC/ Kulubara‐1274). Upon growing, the leaves and flowers were harvested in spring and then milled after being dried at room temperature (25 ± 2°C). After that, 220 g of the dried leaf and flower powder was dissolved in 70% ethyl alcohol solution for 72 hr; the solution was then filtered through filter papers (Whatman filter paper no.42, Millipore. Cat No. 1442125) and dried at room temperature. Finally, after evaporation of alcohol, 30 g of the extract was stored at 4°C (Ahmeda, Zangeneh, & Zangeneh, 2020; Bazm, Khazaei, Ghanbari, & Naseri, 2018; Bazm, Naseri, & Khazaei, 2018).

### 2.2 Cell culture

**FIGURE 1:**
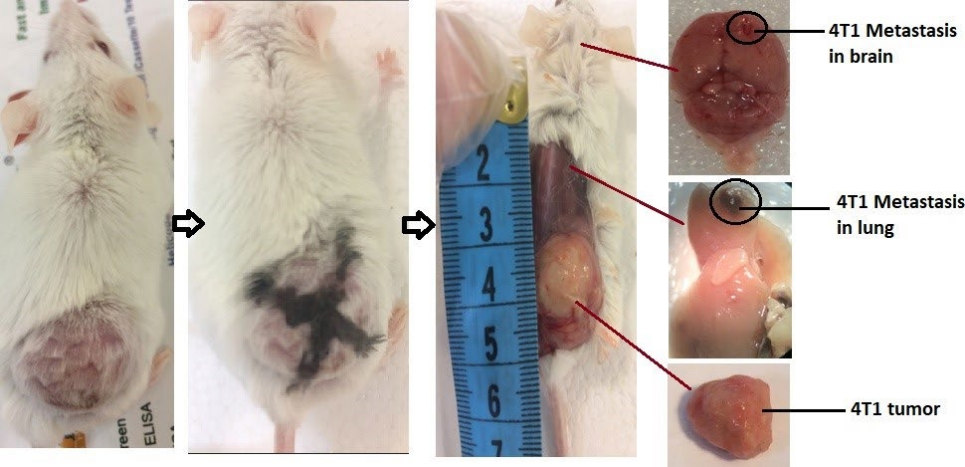
4T1 tumor induction and metastasis in lung and brain in BALB/c mice

The 4T1 mouse BC cell line (ATCC CRL‐2539) was obtained from cell bank (Pasteur Institute). Cells were cultured in RPMI‐1640 medium supplemented with nonessential amino acids, 1% penicillin–streptomycin, and 10% FBS at 37°C and 5% CO_2_ (Sigma). 4T1 cells cultured (>85% viability) in RPMI‐1640 medium were washed with PBS and counted after the treatment of trypsin–EDTA solution (Invitrogen). 1 × 10^6^ cells were diluted in PBS and injected subcutaneously into the right mammary fat pad. After 10 days, the tumors grew and were palpable in mice (70% incidence rate) (Khazaei & Pazhouhi, 2019; Mahdavi et al., 2020) (Figure 1).

### 2.3 Animals and treatment

Fifty‐six female BALB/c mice (*n* = 8/group) were purchased from the Pasteur Institute. All the animals were housed in 12 hr light–dark cycle, at 24 ± 2°C, and 50 ± 10% relative humidity and fed with solid standard pellets and water ad libitum in accordance with the rules of working with laboratory animals. The protocols were approved by the Ethics Committee of Kermanshah University of Medical Sciences (Ethic code: IR.KUMS.REC.1398.359) and performed in line with the guidelines of the Animal Ethics Committee (NIH Publication 80‐23, 1996).

### 2.4 Study groups

The study groups included the following: N: normal group (without 4T1 tumors) receiving 0.5 ml distilled water (DW) by gavage, C−: negative control group treated with 0.5 ml DW by gavage, C+: positive control group receiving a single dose of 5 mg/kg of DOX intravenously (IV), t400t: *T. pratense* extract treatment group receiving 400 mg/kg hydroalcoholic extract of the plant by gavage, and DOX + t: three synergism treatment groups treated with either 100 (t100), 200 (t200), or 400 (t400) mg/kg hydroalcoholic extract of *T. pratense* through gavage plus 5 mg/kg single dose of DOX (IV). In the last three groups, *T. pratense* extract and DOX were administered at 24‐hr intervals at a specified time of day. All the treatments continued for 35 days. *Trifolium pratense* extract and DOX were dissolved in 0.5 ml DW (Akbaribazm et al., 2020a, 2020b; El‐Gendy, 2012).

### 2.5 Cytokines assays

At the end of the study (the day 45 after tumor induction), the animals were sacrificed after IP injection of ketamine (40 mg/kg), and blood samples were taken from the heart. The sera were isolated (12000 *g*for 20 min), stored at −20°C and used for measuring cytokines by commercial enzyme‐linked ELISA kits (IL‐8, Cat. No: 511408; IL‐ 6, Cat. No: 431301; BioLegend) following the manufacturer's instructions. Briefly, a 96‐well plate was precoated with the primary antibodies for overnight at 4°C. After three times washing with PBS, blocking bovine serum albumin was added and incubated for 1 hr. After preparing standard dilutions, the samples were added to the plates. The plates were then rewashed for omitting nonattached antibodies and further incubated with diluted detection antibodies for 30 min. The TMB substrate was added to the plates, yielding a yellow color product. Finally, the absorption was read by a microplate reader (BioTek Instruments) at 450 and 570 nm wavelengths, and the results were reported as pg/dl (Akbari Bazm, Goodarzi, Shahrokhi, & Khazaei, 2020).

### 2.6 Stereological histopathology

**FIGURE 2:**
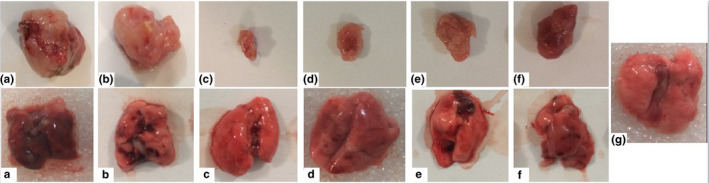
The volume of tumors (top row) and their lung metastasis (bottom row) in C− (A, a), C+ (B, b), t400 (C, c), t200 (D, d), t100 (E, e), t400t (F, f), and normal groups (g)

The lung, brain, and tumor tissues were fixed in 10% formalin for 72 hr after being removed (Figure 2). The primary volumes of the brain and lung tissues were measured by the immersion and cavalier's techniques, respectively, and after estimating the tissues shrinkage rates, isotropic uniform random (IUR) sections were prepared by the orientator method. After tissue processing, paraffin blocks were prepared, 5‐μm sections were cut from the blocks (Leica Microsystems), and the tissue sections were finally stained with Masson's trichrome (tumor tissues and lung), hematoxylin and eosin (H&E) (lung), and methylene blue (brain) (Hagh‐Nazari et al., 2017).

**FIGURE 3:**
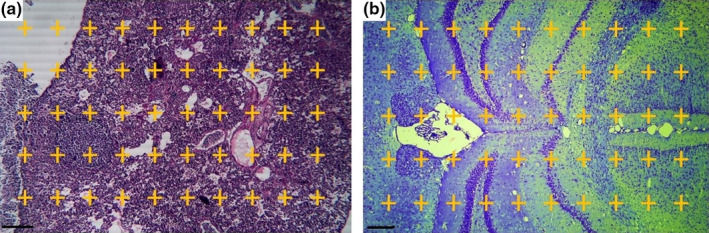
Point probe (50 points) for calculating the volumetric density of lung (a) and brain (b). The total number of points located on each structure is counted in each field of view and is compared to the total number of points in the volume measurement formula: *V*_v_ = ∑*P*_structure_/∑*P*_reference_ (a: lung, H&E, and b: brain, methylene blue staining, scale bar = 300 μm, ×40)

The point probe was used to calculate the relative volume (*V*_v_) of structures in lung (total volume, tissue parenchyma, air parenchyma, vessels, and tumor) and brain (neuronal tissue, vessels, ventricles, and tumor) by using a Nikon light microscopy equipped with Kecam (Kecam Technologies) and TopView software (Version 3.7). The images were processed using Adobe Photoshop CC (Adobe system). Finally, the relative volume was multiplied by the reference volume to calculate the final volume of each structure (Figure 3) (Akbari, Goodarzi, & Tavafi, 2017).

### 2.7 Immunohistochemical (IHC) assay

Cytokeratin 5/6 (CK5/6), a metastatic index of tumors, and GATA binding protein 3 to DNA sequence [A/T] GATA[A/G] (GATA‐3) were assessed by immunohistochemistry (IHC) staining in the lung and brain. The tissues (tumor, brain, and lung) were fixed in 10% formalin, and the paraffin blocks and tissue slides (5 µm) were prepared after tissue processing. Finally, the tissue sections were placed on glass slides and covered with 3‐triethoxysilyl propylamine. The slides were incubated at 65°C for 60 min, then deparaffinized, rehydrated, and incubated at 95°C for 20 min with EDTA–Tris buffer (0.4 g EDTA + 2 g tris dissolved in 1 L of distilled water, PH = 9) for antibody retrieval (heat‐induced epitope retrieval [HIER]). Afterward, the slides were incubated in 3% H_2_O_2_ and blocked with 5% BSA to inactivate endogenous peroxidase and then washed twice in PBS (3 min). Biotinylated rabbit anti‐human CK5/6 IgG monoclonal antibody (clone EP67 + EP24) and mouse anti‐human GATA‐3 monoclonal antibody (clone L50‐823) (Master Diagnostica, CK5/6 Cat. No: MAD‐000651QD‐7 and GATA‐3 Cat. No: MAD‐000632QD–7) (incubation for 60 min at room temperature), streptavidin–horseradish peroxidase (incubation for 30 min as evasion solution), 1, 3‐diaminobenzidine (DAB) tetrahydrochloride (incubation for 10 min), and hematoxylin counterstain were applied. The slides were washed at each step with a washing buffer [Tris buffer (0.6 g tris + 8 g NaCl–Tris dissolved in 1 L distilled water), PH = 7). The results were analyzed using a light microscope (Olympus IX71 microscope) equipped with Kecam (Kecam Technologies), TopView software (version 3.7), and point probe. Randomly chosen fields (10 fields/slide) were photographed at ×400 magnification. The data were presented as mean ± *SD* percentage calculated as CK5/6‐ and GATA‐3‐positive cells/square points (i.e., CK5/6‐ and GATA‐3‐negative cells) × 100 (Laurinavicius et al., 2012).

### 2.8 Quantitative real‐time PCR

#### 2.8.1 RNA extraction and cDNA synthesis

Total RNA was extracted from 40 mg of the tumor tissue using a RNA extraction mini kit (Cat No: FATRK 001, Favorgen Biotech Corp) and TRIzol following the manufacturer's protocol. The quality of the extracted RNA was determined by electrophoresis on 2% agarose gel, and its concentration and purity were assessed using NanoDrop spectrophotometer (BioTeK) by reading A_260_/A_280_ and A_260_/A_230_ nm absorbance ratio. The extracted RNA (1,000 ng) was converted to cDNA using a cDNA synthesis kit (BioFact™ cDNA synthesis kit, Cat.No. BR123‐R10k, BioFact™ RT Series). The reaction mixture included 10 µl 5X RT Reaction Buffer, 1 µl Random Hexamer Primer, 1 µl oligo‐d (T), and 1 µg of total RNA, which was reached to the final volume of 20 µl by adding DEPC water. The reverse transcription was performed at 95°C for 2 min, and then 60°C for 30 s. The enzymatic reaction was stopped by heating at 74°C for 4 min. The cDNA template was stored at −20°C until use (Akbari Bazm et al., 2020).

### 2.9 Real‐time qRT‐PCR assay

The mRNA levels of MMP‐2 and SIRT‐1 were evaluated using High ROX BioFact™ 2X Real‐Time PCR Smart mix SYBR Green PCR Master Mix and Real‐Time PCR lightcycler device (StepOne™ Real‐Time PCR System) based on the manufacturers' protocols. PCR primers were designed by the Oligo software, and the sequences were blasted in the NCBI database (Table 1). GAPDH was used as housekeeping gene. The qRT‐PCR mixture was prepared by mixing 1 μl cDNA, 10 ml SYBR Premix ExTaq II, and 1 μl of each of forward and reverse primers. The final volume of the mixture reached to 20 μl by adding deionized water. PCR program for determining mRNAs expressions was 95°C for 1 min (denaturation) followed by 95°C for 30 s and 42 cycles at 60°C for 1 min (annealing) and 72°C for 1 min (extension). All qRT‐PCRs were carried out in duplicate. Gene expression levels were determined using the Ct (2^−ΔΔt^) method.

$$\Delta \Delta \text{CT}\;=\;\left[{\left({\text{mCT}}_{\text{target}}\;‐\;{\text{mCT}}_{\text{reference}}\right)}_{\text{test}\;\text{sample}}\;‐\;{\left({\text{mCT}}_{\text{target}}\;‐\;{\text{mCT}}_{\text{reference}}\right)}_{\text{control}\;\text{sample}}\right]$$

**TABLE 1:** Primer sequences

Gene	Sequences
GAPDH	F: 5‐AACTTTGGCATTGTGGAAGG‐3 R: 5‐ ACACATTGGGGGTAGGAACA‐3
MMP‐2	F: 5‐CCCCGATGCTGATACTGA‐3 R: 5‐CTGTCCGCCAAATAAACC‐3
SIRT‐1	F: 5‐AGCTGGATGATATGACGC‐3 R: 5‐CCCACAGGAGACAGAAAC‐3

Finally, the expression levels of the target genes were determined as 2^−∆∆CT^ (Akbari Bazm et al., 2020).

### 2.10 Statistical analysis

All statistical analyses were conducted in SPSS 16.0 applying one‐way ANOVA and post hoc Duncan's test at the significance level of *p* < .05. Normality of the data was assessed by the Kolmogorov–Smirnov test (*p* > .05). The values were presented as mean ± *SD*.

## 3 RESULTS

### 3.1 Serum cytokine levels

**FIGURE 4:**
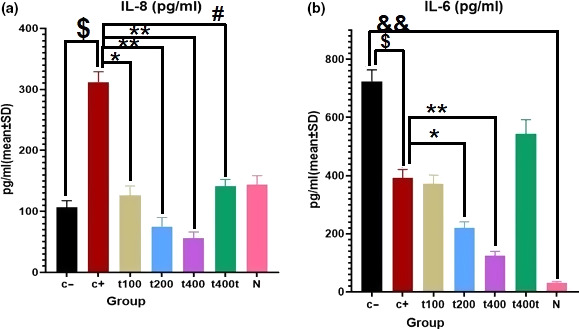
Comparison of serum cytokine levels of (a) IL‐6 and (b) IL‐8 (pg/ml) in negative control (C−), positive control (DOX, C+), the extract groups (t100, t200, and t400) and t400t (mean ± *SD*). ^$^(*p* < .05) statistically significant between DOX and negative control groups (C−), ^#^(*p* < .05) statistically significant between t400 and DOX groups, *(*p* < .05) and **(*p* < .01) statistically significant between treatment and DOX groups, and ^&&^(*p* < .01) statistically significant between C− and normal groups

IL‐6 significantly (*p* = .001) increased in C− group compared with normal (N) group. Treatment with DOX alone significantly decreased the serum level of IL‐6 (*p* = .021) and significantly increased the level of IL‐8 (*p* = .012) compared with C− group. In the groups treated with the extract, there were significant declines in IL‐8 level (*p* = .013) in the DOX + t100 and IL‐8 (*p* = .006) and IL‐6 (*p* = .011) levels in the DOX + t200 group. Also, IL‐8 (*p* = .002) and IL‐6 (*p* = .007) levels significantly decreased in DOX + t400 group compared with DOX group. Finally, t400t group showed significant reduction in IL‐8 level (*p* = .011) compared with DOX group (Figure 4).

### 3.2 SIRT‐1 and MMP‐2 mRNA expressions

**FIGURE 5:**
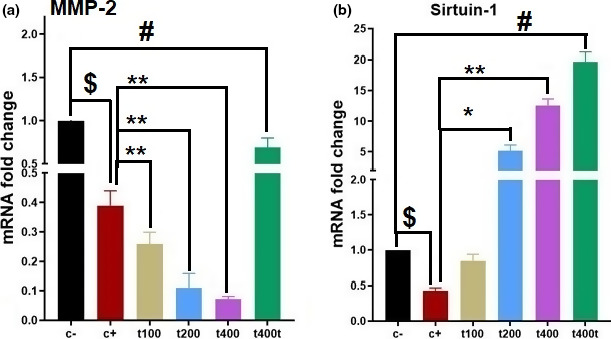
The effect of *Trifolium pratense* on (a) MMP‐2, (b) sirtuin‐1 (SIRT‐1) gene expression of tumors tissue in negative control (C−), positive control (DOX, C+), the extract groups (t100, t200, and t400) and t400t (mean ± *SD*). ^$^(*p* < .05) statistically significant between DOX and negative control groups, ^#^(*p* < .05) statistically significant between t400t and negative control groups, and *(*p* < .05) and **(*p* < .01) statistically significant between treatment and DOX groups

To evaluate the metastatic activity of tumors, the mRNA expression levels of metastasis‐related genes (MMP‐2 and SIRT‐1) were measured. DOX treatment significantly down‐regulated SIRT‐1 (*p* = .021) and MMP‐2 (*p* = .016) mRNA levels compared with the C− group. Exposition to DOX + 100 mg/kg of the extract significantly down‐regulated MMP‐2 (*p* = .007) while DOX + 200 mg/kg group showed a significant boost in mRNA level of SIRT‐1 (*p* = .033) and a decline in MMP‐2 (*p* = .003) level. Treatment with DOX + 400 mg/kg of the extract significantly up‐regulated the mRNA expression of SIRT‐1 (*p* = .005) and down‐regulated that of MMP‐2 compared with the DOX group. In addition, treatment with 400 mg/kg of *T. pratense* extract alone significantly up‐regulated the mRNA level of SIRT‐1 (*p* = .0006) while down‐regulated MMP‐2 (*p* = .042) compared with C− group (Figure 5).

### 3.3 CK5/6 and GATA‐3 expressions in tumor, lung, and brain tissues

**FIGURE 6:**
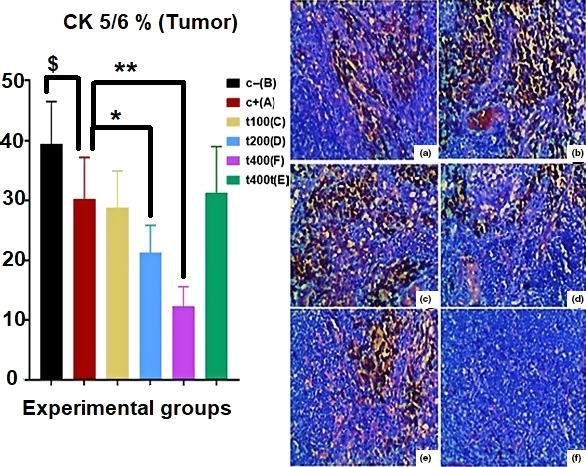
The effect of *Trifolium pratense* on CK 5/6 expression (CK 5/6‐positive cells) of tumors tissue in positive control (DOX, a), negative control (C−, b), and the extract groups (t100 [c], t200 [d], and t400 [e]) and t400t (f) (mean ± *SD*). ^$^(*p* < .05) statistically significant between DOX and negative control groups, *(*p* < .05) and **(*p* < .01) statistically significant between treatment and DOX groups

**FIGURE 7:**
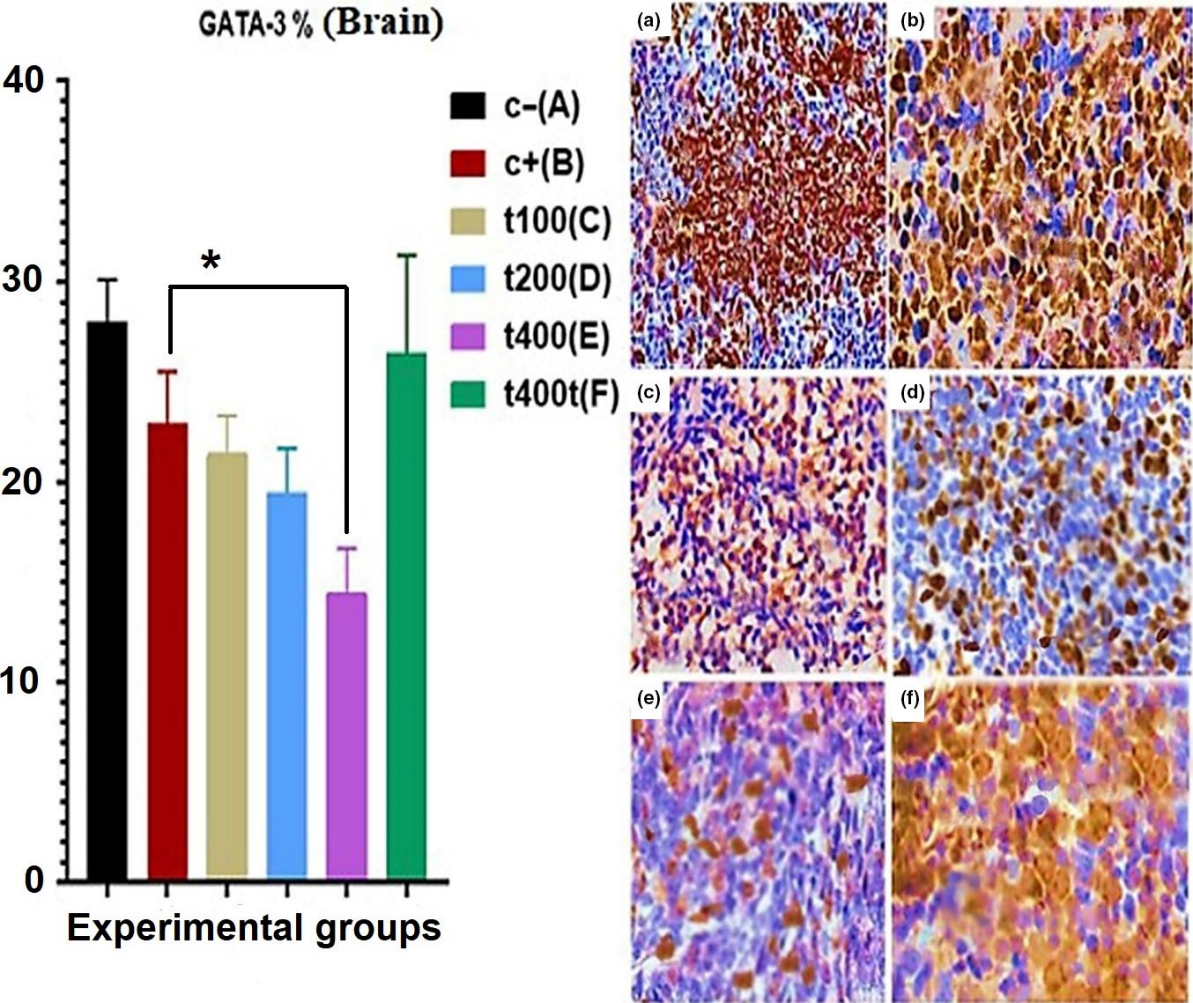
The effect of *Trifolium pratense* on GATA‐3 expression (GATA‐3‐positive cells) of brain tissue in negative control (C−, a), positive control (DOX, b), and the extract groups [t100 (c), t200 (d), and t400 (e)] and t400t (f) (mean ± *SD*). *(*p* < .05) statistically significant between treatment and DOX groups

**FIGURE 8:**
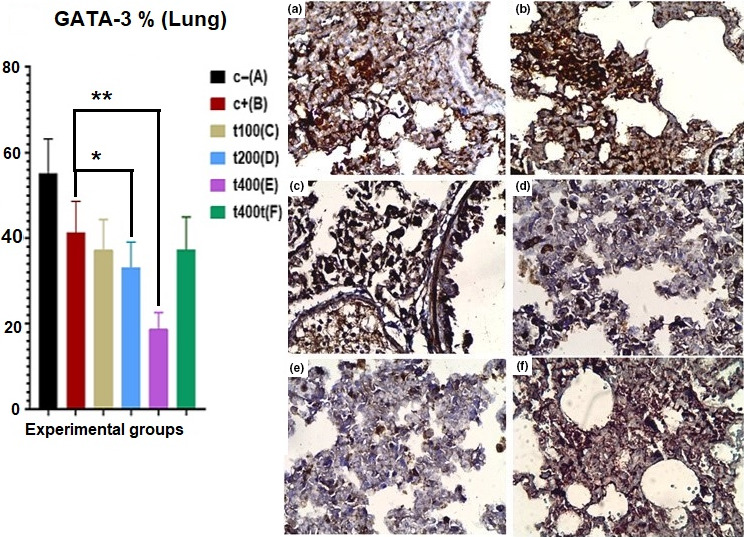
The effect of *Trifolium pratense* on GATA‐3 expression (GATA‐3‐positive cells) of lung tissue in negative control (C−, a), positive control (DOX, b), and the extract groups [t100 (c), t200 (d), and t400 (e)] and t400t (f) (mean ± *SD*). *(*p* < .05) and **(*p* < .01) statistically significant between treatment and DOX groups

The percent of cells expressing phosphorylated CK 5/6 in tumor tissues significantly decreased (*p* = .043) in the DOX group as compared to the C− group. The DOX + 200 mg/kg (*p* = .032) and DOX + 400 mg/kg (*p* = .018) groups showed significant reductions in the expression of CK5/6 as compared to the DOX group (Figure 6). After calculating the percentage of cells showing positivity for phosphorylated GATA‐3 in the lung and brain tissues, it was found that treatment with DOX + 200 mg/kg of the extract significantly (*p* = .026) decreased GATA‐3‐positive cells in the lung. Likewise, the DOX + 400 mg/kg group showed significant declines in GATA‐3 positivity both in the lung (*p* = .011) and in the brain (*p* = .032) tissues compared with the C− group (Figures 7 and 8).

### 3.4 Histopathological findings

**FIGURE 9:**
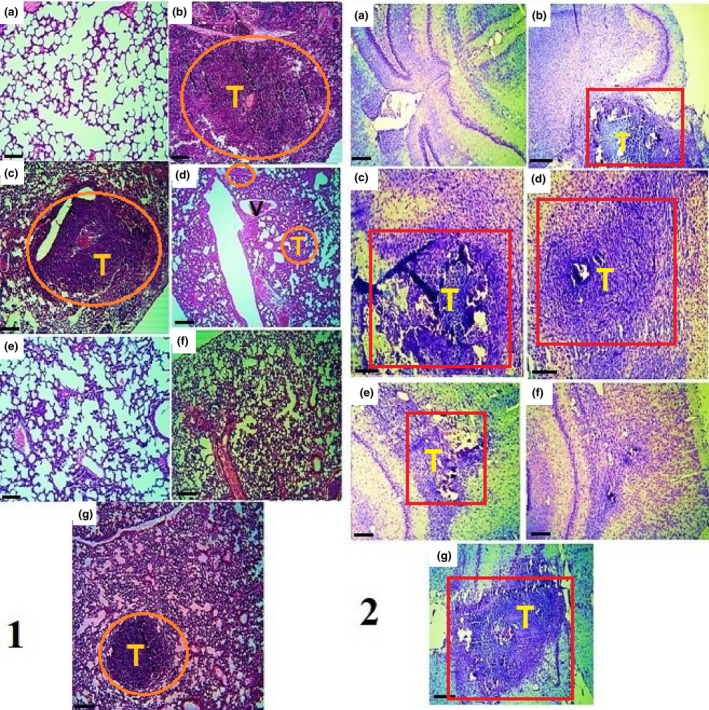
Histopathological examination in lung (1) and brain (2) in normal tissue (a), negative control (C−, b), positive control (DOX, c), and the extract groups (t100 [d], t200 [e], and t400 [f]) and t400t (g). Red square (metastatic brain tumor), and orange circle (metastatic lung tumor); T: tumor and V: vessel (1: H&E and 2: methylene blue staining, scale bar = 300 μm, ×40)

After measuring the final volumes of the lung and brain tissues, stereological parameters were estimated. The final volumes of the brain (*p* = .034) and vessels (*p* = .026) significantly increased in the C− group in comparison with the normal (N) group. However, the volumes of neurons (*p* = .034) and ventricles (*p* = .039) of the brain significantly decreased in the C− group compared with the normal group. Furthermore, the final volume of the brain significantly (*p* = .026) decreased in the DOX + t400 group compared with the DOX group. Regarding tumor volume, significant reductions were recorded in the DOX + t200 (*p* = .012) and DOX + t400 (*p* = .006) groups compared with the DOX group. Also, the brain neuronal volume was significantly higher in the DOX + t200 (*p* = .036) and DOX + t400 (*p* = .029) groups than the DOX group. On the other hand, the volume of ventricles was significantly lower in the DOX in comparison with the normal group (*p* = .041) while it was significantly higher in the DOX + t400 compared with the DOX group (*p* = .034) [Figures 9(2) and 10a].

**FIGURE 10:**
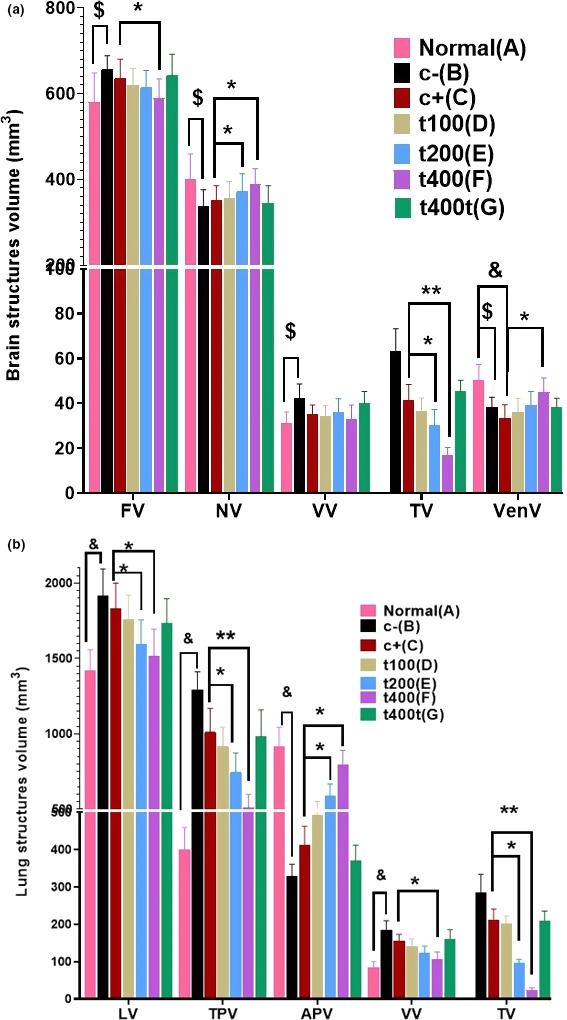
The effect of DOX and *Trifolium pratense* on stereological parameters in (a) brain (nervous tissue, vessels, ventricles, and tumor) and (b) lung (total volume, tissue parenchyma, air parenchyma, vessels, and tumor) in normal tissue (a), negative control (C−, b), positive control (DOX, c), and the extract groups (t100 [d], t200 [e], and t400 [f]) and t400t (g) (mean ± *SD*). ^&^(*p* < .05) statistically significant between negative and normal control groups, ^$^(*p* < .05) statistically significant between DOX and normal groups, *(*p* < .05) and **(*p* < .01) statistically significant between treatment and DOX groups. (a) Final volume (FV), nervous volume (NV), vessels volume (VV), tumor volume (TV), and ventricle volume (VenV). (b) Lung volume (LV), tissue parenchyma volume (TPV), air parenchyma volume (APV), vessels volume (VV), and tumor volume (TV)

In comparison with the DOX group, the final volumes of the lung (*p* = .031 and .019), parenchyma (interstitial) (*p* = .022 and .008), and tumors (*p* = .011 and .003) were significantly lower in the DOX + t200 and DOX + t400 groups, respectively. On the other hand, the volume of lung air parenchyma significantly increased in the DOX + t200 (*p* = .027) and DOX + t400 (*p* = .011) groups compared with the DOX group [Figures 9(1) and 10b].

## 4 DISCUSSION

In this study, 4T1 breast cancer‐bearing mice were used to evaluate the efficacy of *T. pratense* hydroalcoholic extract in vivo. The 4T1 cancer is a subtype of TNBCs, lacking hormone receptors (ER and PR) and HER_2_^‐^ and therefore are resistant to hormone replacing therapy (HRT) and have high potency for metastasis to lungs, the brain, and other organs of the body. In this study, we showed that red clover extract in combination with DOX reduced the metastasis of these cells to the lung and brain and also increased the viability of tumor‐bearing mice.

The synergistic effect of red clover extract with DOX reduced serum levels of IL‐6 and IL‐8 inflammatory cytokines compared with the control groups treated with either DOX or red clover extract alone. The IL‐6/JAK/STAT3 pathway modulates the expressions of several genes involved in the proliferation, survival, and transformation of breast cancer cells. The elevated serum level of IL‐6 in 4T1‐bearing animal models of BC has been shown to attenuate by DOX treatment (Johnson, O'Keefe, & Grandis, 2018). According to the present study, it seems that red clover extract can decrease the proliferation and viability of cancer cells by decreasing serum levels of inflammatory cytokines, especially IL‐6.

The role of IL‐8 is important in the migration and invasion of cancerous cells and the progression and metastasis of tumors. A high level of IL‐8 in TNBCs is associated with more invasive behavior of these cells. Also, the expressions of adhesion molecules such as fibronectin on human BC cells are affected by the act of IL‐8 (Todorović‐Raković & Milovanović, 2013). IL‐8 induces the upregulation of integrin β_3_ and increases the invasive potential of BC cells via activating the PI3K/Akt pathway and subsequently NF‐κB. In turn, NF‐κB plays an important role in inducing IL‐8 gene transcription via several signaling mediators such as LPS, TNF‐α, and oxidative stress (Shao et al., 2015). The IL‐8/PI3K/Akt/NF‐κB/integrin β3 axis has been considered as a therapeutic target to reduce the growth and progression of metastatic breast tumors. It has been noted that oxidative stress significantly motivates the migratory potential of TNBCs in part through inducing the secretion of promigratory IL‐8 chemokine and activating the Erk signaling pathway (Merchant et al., 2017).

There has been an inverse relationship between IL‐8 level and ER expression; however, the mechanism of action of IL‐8 in TNBCs is largely unknown. In TNBCs, the expressions of ER and growth factors are affected by the mitogen‐activated protein kinase (MAPK) cascade which is a part of IL‐8 signaling pathway. During the progression of breast tumors, inactivating ERs upregulates IL‐8 level (Freund et al., 2003). Finally, suppressing IL‐8 as an angiogenic and tumorigenic cytokine can be therapeutically important for inhibiting the invasion and metastasis of breast tumors. In the present study, red clover extract decreased the angiogenesis and metastasis of BC cells by decreasing the serum level of IL‐8. The compounds in this plant appear to do this by affecting pathways related to ERs, as well as modulating the expression of adhesion molecules and secretion of angiogenic factors such as VEGF.

Secondary to chemotherapy‐induced cytokine release and systemic inflammation, DOX causes multiorgan damages in cancer patients. It can increase the levels of pro‐inflammatory interleukins such as IL‐8 and IL‐6 (Elsea, Roberts, Druker, & Wood, 2008). However, these changes depend on the types of cancer cells, the dose of DOX (usually needs a dose >5 mg/kg), and receiving complementary drugs (Buoncervello et al., 2019). In human small‐cell lung cancer cell lines, DOX increased the production of IL‐8. In the present study, DOX increased the level of IL‐8 inflammatory cytokine as well. In another study, isoflavones and their metabolites were found to decrease IL‐6 level in mouse models of LPS‐induced inflammation (Kao, Wu, Hung, Wu, & Chen, 2007). In human monocytic THP‐1 cells, green soybean extract suppressed the LPS‐induced production of IL‐6, IL‐12, and TNF‐α (Tanaka et al., 2014). These results are in line with ours showing that red clover isoflavones reduced serum levels of IL‐6 and IL‐8 inflammatory cytokines.

In animal models and humans, oxidative stress, by mediating cellular signal transduction pathways, plays a major role in tumor initiation, survival, and metastasis (Trush & Kensler, 1991). MMPs cleave the basement membrane components and extracellular matrix proteins such as type IV collagen and are key enzymes for metastasis and invasion of breast tumor cells. MMP‐2 is activated by the reaction of ROS with the thiol groups of the protease catalytic domain. Also, studies showed that H_2_O_2_ activated MMP‐2 and increased cell invasion in fibroblastic cells (Pelicano, Carney, & Huang, 2004). Furthermore, in vivo studies showed that the administration of either H_2_O_2_ or an oxidative stress‐producing drug such as DOX activated angiogenesis in cancerous tissues (Monte, Davel, & de Lustig, 1997).

Overexpressed MMP‐2 and MMP‐9 in BC cells facilitate the degradation of type IV collagen, increasing the metastasis and invasion of cancer cells. Elevated expression of these enzymes has been associated with poor prognosis in BC (Webb et al., 2017). Thus, suppressing MMP‐2 and MMP‐9 can be a critical step to inhibit metastasis in cancer. A study showed that formononetin inhibited the proliferation of BC cells by promoting inhibitory effects on the IGF1/PI3K/Akt pathway (Chen, Zeng, Xin, Huang, & Chen, 2011). Also, this isoflavone at the doses of 20 and 40 μmol/L reduced the expressions of MMP‐2 and MMP‐9 via PI3K/Akt signaling pathway in TNBC cell lines (MDA‐MB‐231 and 4T1) and inhibited the invasiveness of these BC cells (Zhou et al., 2014).

Zhou et al. (2019) showed that SIRT‐1 suppressed hepatocellular carcinoma cell line growth and metastasis via activating the NF‐κB pathway and promoting M1 macrophage polarization (Zhou et al., 2019). In BC metastasis, the SIRT1‐PRRX1‐KLF4 axis is the core circuitry and controls the expressions and activities of EMT and cell adhesion molecules, such as E‐cadherin, as well as MMPs. The activity of SIRT1‐PRRX1 axis has been associated with lung metastasis of BC cells (Shi et al., 2018). In the present study, we showed that DOX and *T. pratense* extract negatively regulated the expression of SIRT‐1 mRNA. While DOX down‐regulated the expression of SIRT‐1, red clover extract significantly increased its expression at the doses of 200 and 400 mg/kg. It can be concluded that isoflavones present in *T. pratense* extract can compensate for the negative effects of DOX by up‐regulating the expression of SIRT‐1 and therefore suppressing the metastasis of BC cells.

GATA‐3 regulates EMT‐associated transcription factors such as the slug, snail, and twist1 that play key roles in EMT. GATA‐3 labeling has been demonstrated to be highly specific for identifying the metastasis of TNBC breast carcinomas in distant sites (e.g., lung and brain) (Sangoi, Shrestha, Yang, Mego, & Beck, 2016). In a study by Sangoi et al., 2016, the sensitivity of GATA‐3 to detect metastasis in TNBC was reported as 95%, and Miettinen et al. (2014) also noted the sensitivities of 92% and 96% in primary and metastatic TNBC, respectively (Miettinen et al., 2014). Thus, GATA‐3 presents an appropriate marker for identifying the TNBC subset of breast cancers. In the present study, DOX and *T. pratense* extract synergistically reduced GATA‐3‐positive cells in brain and lung tissues. DOX along with folate‐conjugated pH‐sensitive polymeric micelles also inhibited tumor growth and the metastasis of 4T1 cells to lung and the heart in murine BC model (Gao, Tian, Hu, Park, & Bae, 2011). In our study, *T. pratense* extract at all the assessed doses (100, 200, and 400 mg/kg) reduced MMP‐2 expression and ultimately inhibited the metastasis of GATA‐3‐positive cancer cells to the brain and lung.

For the identification and classification of the basal subgroup of TNBCs, CK5/6 is the most useful and important marker. Based on the growth phase and metastasis rate of TNBC tumors, CK5/6 expression varies from 24% to 72% (Dogu et al., 2010). Independent of hormonal HER2/neu status, T stage, and tumor grade, CK5/6‐positive BCs have poor prognosis. There has been a positive correlation between TNBC tumor size and nodal metastasis in CK5/6‐positive tumors (Hashmi et al., 2018; Hemmati, Zamenian, et al., 2020). BCs with brain and lung metastases are more likely to be ER^‐^ and PR^‐^ and express basal CK5/6. In comparison with those with the luminal subtype, patients with TNBC have higher sensitivity to DOX and higher rates of pathologic complete response regarding immunohistochemical positivity for Ki‐67 and CK5/6 (Carey et al., 2007). Our study also showed that red clover extract and DOX synergistically reduced the number of CK5/6‐positive cells in tumor tissues.

## 5 CONCLUSION

Our results present the first evidence on the antimetastasis effects of *T. pratense* hydroalcoholic extract in synergy with DOX as demonstrated by reduced metastasis in 4T1‐bearing BALB/c mice with orthotopic BC tumors in vivo. The herb–drug interaction between *T. pratense* extract and conventional anticancer agents was also examined. Our observation holds promise for further studies to examine the chemotherapeutic efficacy of *T. pratense* extract as a potential anticancer and antimetastatic supplement in TNBC.

## CONFLICT OF INTEREST

The authors declare that there is no conflict of interest.

## ETHICAL APPROVAL

The overall process of research with animals was performed under the supervision of the Ethics Committee of Kermanshah University of Medical Sciences (Ethic code: IR.KUMS.REC.1398.359) in line with the protocol of the Animal Ethics Committee (NIH Publication 80‐23, 1996).

## CONSENT FOR PUBLICATION

Not applicable.

## Data Availability

All data generated or analyzed during this study are included in this published article.
